# How Do Primary Care Physicians Perceive the Role of Nurses in Quality Measurement and Improvement? The Israeli Story

**DOI:** 10.3389/fpubh.2016.00124

**Published:** 2016-06-22

**Authors:** Rachel Nissanholtz-Gannot, Dorit Goldman, Bruce Rosen, Calanit Kay, Rachel Wilf-Miron

**Affiliations:** ^1^Department of Health Management, Ariel University, University Hill, Ariel, Israel; ^2^Smokler Centre for Health Policy Research, Myers-JDC-Brookdale Institute, Jerusalem, Israel; ^3^Meuhedet Health Services, Tel-Aviv, Israel; ^4^Community Health Services, Clalit Health Services, Tel Aviv, Israel; ^5^The Gertner Institute for Epidemiology and Health Policy Research, Tel Aviv, Israel; ^6^The School of Public Health, Sackler Faculty of Medicine, Tel Aviv University, Tel Aviv, Israel

**Keywords:** attitudes, nurses, quality improvement, quality monitoring

## Abstract

**Background:**

Israel has boasted a highly effective national quality monitoring program for community-based health services since 2004. The program involves ongoing monitoring of the quality of selected services provided by Israeli health plans and includes approximately 70 indicators.

**Objective:**

To analyze Israeli primary care physicians’ (PCPs) perceptions of nurses’ roles in the national quality monitoring program and their contribution to improving health-care quality.

**Design:**

A cross sectional survey using self-reported questionnaire.

**Setting:**

Four Israeli health plans, covering 100% of the Israeli population.

**Participants:**

A representative sample of 1,000 Israeli PCPs. Response rate of 69% (605 out of the 884 physicians who met the study criteria).

**Methods:**

A questionnaire combined with closed questions on the attitudes and behaviors of the physicians regarding nurses’ involvement in quality monitoring and open questions about the changes that had made in their practice as a result of the quality monitoring program.

**Results:**

Most respondents (74%) agreed that nurses contribute to practice quality and share responsibility for improving quality measures. Physicians who felt that quality monitoring improved the quality of care and those who supported the program were more likely to consider that nurses shared responsibility for the quality of care. However, in open-ended questions about the changes they made in their practices as a result of the program, they made minimal reference to the importance of nurses and their contribution to improved quality indicators.

**Conclusion:**

There was a disparity between the closed-ended and open-ended questions regarding the way physicians depicted the role of nurses in quality monitoring and improvement. This disparity may be due to the fact that physicians do not yet fully appreciate the growing involvement of nurses in these areas.

## Introduction

Over the past two decades, monitoring and public reporting on the quality of medical care have attracted increasing attention (1, 2). The first national monitoring system in the United States was the health-care effectiveness data and information set (HEDIS), which was adopted by the National Committee for Quality Assurance in 1996. Subsequently, monitoring systems have been instituted in other countries (3–5). In Israel, the Ministry of Health has implemented a national quality monitoring program for community-based health services since 2004. The program uses some 40 quality measures to monitor 6 clinical domains that are relevant mainly to primary care: immunization, early detection of cancer, detection and control for cardiovascular risk factors, as well as asthma and diabetes care. All four of the Israeli health plans participate voluntarily in the quality monitoring program, which in effect covers the entire population of Israel, since national health insurance is provided by law and all residents of the state are insured through the health plan of their choice. From 2004 to 2010, a steady improvement was demonstrated at the national level in most of the measures monitored by the national program (6, 7).

In line with the increasing efforts to improve the quality of health care, the involvement of nurses in quality improvement has been changing in many countries (8–11) and is being tested (12). Increased numbers of chronic patients, a shortage of medical manpower, and structural changes in health systems have all pushed the nursing profession to the forefront of medical care in general (13, 14) and quality improvement in particular (9, 15). Today, nurses in many settings around the world are responsible for monitoring patients’ health and improving the quality of the care provided to them (16, 17). In this changing world, nurses have responsibility for immunization, treatment of complex wounds (18), tutorials for a healthy lifestyle (19), and management of care for chronic patients (20, 21). Arguably, nurses have become key partners in efforts by the medical professions to promote community-based quality of care.

Indeed, interviews with managers in the Israeli health system before this study had already indicated that nurses play a meaningful role in many efforts aimed at improving the quality of medical care, mainly through engaging in proactive work with patients, providing explanations and guidelines on medication and a healthy lifestyle, and implementing technological tools for monitoring chronic patients (22). However, not all physicians around the world appeared to support the ever-increasing autonomy and authority of the nursing profession, and there was evidence that some of them would prefer to assign more traditional tasks to nurses (23, 24). Therefore, it was not clear to what extent the emerging role of nurses had been accepted by physicians in Israel.

The objective of this study was to analyze the attitudes of Israeli primary care physicians (PCPs) to the role of nurses in improving clinical performance and their contribution to the quality of health care.

## Materials and Methods

### Study Population

A survey was conducted from August to December 2010 among a representative sample of 1,000 PCPs (excluding pediatricians) employed by the four health plans in Israel. Details of the characteristics of the study population, the sampling method, and methodology appear in an article by Nissanholtz-Gannot et al. (25).

Of all the 1,000 physicians in the sample, 884 met the study criteria. The questionnaire was mailed to them, and they were given the choice of responding by post (53%), telephone (33%), or e-mail (13%). Altogether, 605 physicians (69% of the sample) responded to the questionnaire.

### Questionnaire

The questionnaire included questions addressing the attitudes and behaviors of the physicians regarding nurses’ involvement in quality monitoring and its impact on medical care. Two closed questions directly addressed the perceived contribution of collaboration with nurses: “How much do the nurses actually contribute to the quality of your practice, as reflected in the monitoring?” and “To what extent do you consider that the nurses in your health plan share the responsibility for improving the quality of care as measured by the quality indicators?” The responses were on a scale of 1 (to a very great extent) to 6 (not at all). The physicians were also asked to rank their agreement with a series of statements describing their own level of responsibility and that of the nurses for improving the quality measures. Finally, they were asked open questions about the changes they had made in their practice and those made by their health plan as a result of the quality monitoring program. We expected them to mention the role of nurses in their responses to these questions.

The independent variables included: age, sex, medical specialty, form of employment, and main type of practice. The dependent variables included the physicians’ attitudes to the nurses’ contribution to quality and their responsibility for the quality monitoring indicators.

### Analysis

The analysis used the complex samples option of SPSS (SPSS for Windows, Version 13.0.1; SPSS Inc., Chicago, IL, USA). To analyze the responses to the open questions, we used the SPSS function that permits coding of multiple responses and quantitative assessment of the frequency of each response. For the open questions, we used a simple computer program based on access, which allows the user to record and analyze the full set of answers to a given question. The answers to all the open questions were coded by category to create the coding book for the questionnaire and subsequently were transferred to SPSS for quantitative analysis. This method allows the researchers to return to an open question and use the explanations provided and even to cite responses and expand the parameters. The final form in which the data were presented included transforming the data into quantitative data and presenting them in tables showing the distribution of the answers.

### Ethics Review

In keeping with Israel’s Ministry of Health regulations, the researchers applied to the Israel Institute for Health Policy Research (which supported the research) and were given an exemption from seeking approval by an ethics committee. The reasons for the exemption were as follows.

1. The main purpose of the study was to examine physicians’ attitudes toward Israel’s measurement program.
2. No clinical information was gathered in the study, which focused solely on the physicians’ attitudes to their work.
3. The study was conducted under the auspices of the Myers-JDC-Brookdale Institute of research and not by a medical facility.

Subsequently, all the respondents (the primary care doctors), received an explanation about the goals of the study, including the fact that they had the right not to respond to the questionnaire, without penalty. It was clearly stated at the beginning of the questionnaire that filling out the questionnaire and returning it to the researchers constituted informed consent to participation in the study.

## Results

### Characteristics of the Sample

There were differences in both the personal characteristics of the participants (age, gender, nationality, and country of origin) and in their professional characteristics (specialty and type of employment – Table 1). As expected of a sample of PCPs, only a small percentage of the respondents (4%) replied that their main practice was not in the community.

**Table 1:** **Distribution of respondents by key personal and professional characteristics (percent)**.

**Personal characteristics**		**Professional characteristics**	
**Age**		**Specialty**	
≤44	26	Family medicine	43
45–60	55	Internal medicine/other	19
>60	19	Not board certified	38
**Sex**		**Form of employment**
Female	44	Salaried only	48
Male	56	Independent only	25
		Both salaried and self-employed	26
**Population group**	**Main type of practice**
Non-Jews	24	Primary care physician	96
Jews	76	Specialist	4
**Country of birth**
Outside of Israel	60
Israel	40

The characteristics of the study population were compared only with those of the physicians in Israel’s largest health plan, which serves 53% of the Israeli population, because comparable data were not available from the other three health plans. The study population was found to be similar in terms of gender and form of employment (salaried vs. self-employed) to the largest plan; the sample included more young physicians, up to age 44 (33% in the sample vs. 21% in the largest health plan), and board-certified specialists (73 vs. 60%).

### Perceived Involvement of Nurses in Improving Quality Measures

**Figure 1:**
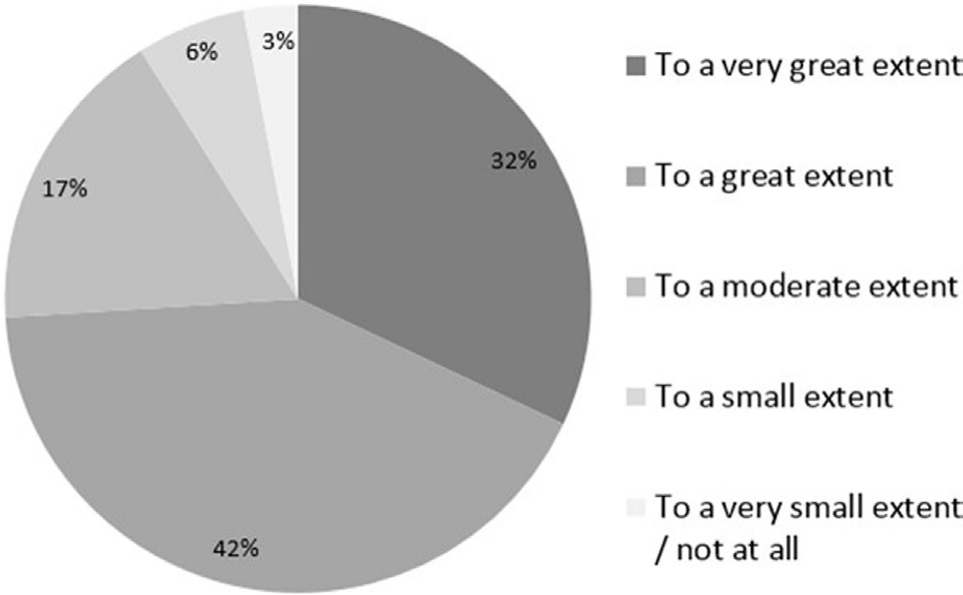
**Physicians’ perceptions of the extent to which responsibility for improving quality measures is shared between physicians and nurses**.

Most physicians (74%) considered that the health plan nurses shared responsibility for improving the quality of medical care to a great or very great extent. Only 3% felt that the nurses shared responsibility to a very small extent or not at all (Figure 1).

The physicians were asked (open-ended questions) what the health plan could do to help them improve the measures. Fifty percent of the respondents replied to this question; 25% of them mentioned nurses in their answers (e.g., “the health plan should add more nurses to the clinics”) while an additional 14% spoke of adding manpower without specific reference to nursing staff. When asked what the main change they would make to the program would be, 7% of those who replied (46% replied to this question) related to nurses (e.g., “the nurses and I work together as a team”). Some physicians also mentioned nurses when asked about the changes that they themselves had made in clinical domains in their practices. For instance, of the 40% responding about diabetes, 7% related to the work with nurses; of the 41% responding about immunizations, only 6% related to nurses.

As indicated in Table 2, most respondents agreed or strongly agreed with various statements to the effect that nurses shared responsibility for improving the quality measures.

**Table 2:** **Perceived levels of responsibility of physicians and nurses for improving quality measures (percent)**.

	**Strongly agree**	**Agree**	**Neutral**	**Disagree**	**Strongly disagree**
The nurses and I share full responsibility for performance on all measures	34	46	13	5	2
The responsibility for performance on all measures is mine and the nurses are supposed to assist me to a great extent	18	48	19	10	5
The responsibility for performance on all measures is mine and the nurses are supposed to assist me to a certain extent	8	22	42	21	7
The responsibility for performance on all measures is mine and the nurses are not supposed to assist me	2	5	8	47	39

### Perceived Contribution to Quality

**Figure 2:**
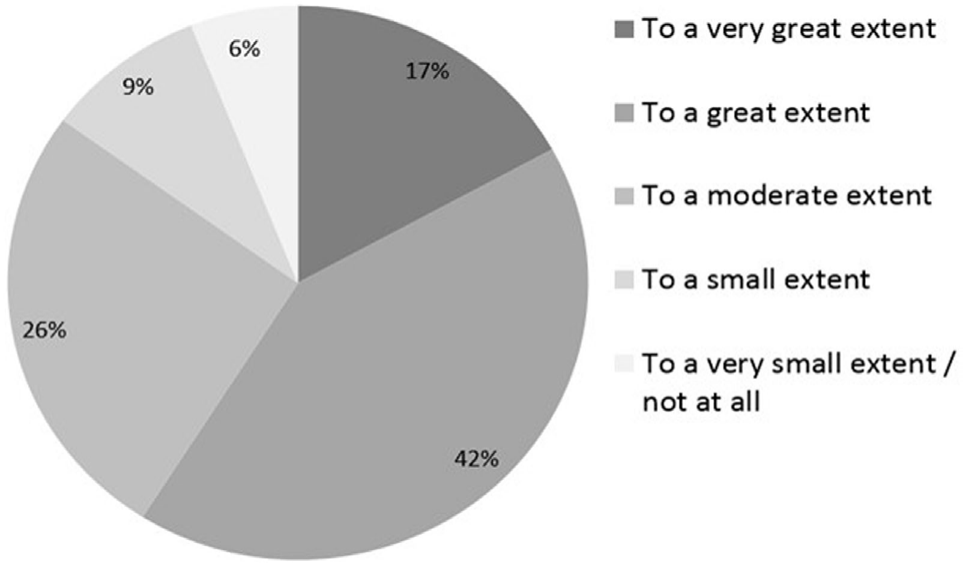
**Physicians’ perceptions of the extent to which the nurses contribute to quality**.

Most physicians (59%) felt that nurses contributed to the quality of their practice to a great or very great extent, while an additional 26% considered their contribution moderate (overall 74%) (Figure 2).

### Correlates and Predictors of Physicians’ Perceptions

A bivariate analysis revealed that the physicians’ characteristics had a minor impact on their perceptions as to the extent that the nurses contributed to quality improvement. The variables of age, nationality, gender, and specialization did not affect their perceptions of nurses. The Israeli-born physicians considered that nurses shared responsibility for the quality of medical care to a greater extent than the non-Israeli-born physicians (80 and 71%, respectively), while salaried physicians tended to regard nurses as sharing responsibility more than the self-employed did (77 and 72%, respectively). Despite the differences among occupational or demographic subgroups, it should be noted that the majority of physicians believed that nurses shared in the process of improving the quality of medical care.

Table 3 presents the results of the logistic regression conducted to examine the relationship between the physicians’ perceptions of the nurses’ contribution to improving the quality of care and their characteristics. The Jewish physicians perceived that nurses contributed to improving medical care 1.5 times more than the non-Jewish physicians; salaried physicians shared this view twice as much as the self-employed, Israeli-born physicians also shared this view 1.8 times as much than physicians who were born abroad, male physicians perceived it lesser than female physicians, and board-certified physicians, no matter what was their specialty, perceived it less than not board-certified physicians. All these differences were statistically significant.

**Table 3:** **Logistic regression of physicians’ perceptions of the nurses’ contribution to improving the quality of medical care**.

**Perceive that “Nurses share responsibility to a great/very great extent”**	
Total	
Age (reference group: age <45)	
45–60	0.887
>60	**0.777**
Born in Israel	**1.869**
Jewish	**1.508**
Male	**0.788**
Board certification (reference group: not board certified)	
Family medicine	**0.751**
Internal medicine/other	0.840
Works primarily as a specialist	
Form of employment (reference group: independent only)	1.084
Salaried only	**2.070**
Both salaried and independent physicians	**1.904**

*Significant findings <0.05 appear in bold*.

We also conducted a logistic regression to examine the relationship between the support expressed by the physicians for continuation of the program and their belief that the measures contributed to improving medical care, on the one hand, and their perception of nurses as being responsible for improving quality, on the other hand. The physicians who felt that the monitoring improved the quality of medical care perceived nurses to share responsibility more than those who did not believe that it did (79 vs. 65%, respectively). Similarly, physicians supporting continuation of the program also believed that nurses shared responsibility for the quality of medical care more than physicians who did not support its continuation (79 vs. 65%, respectively). At the same time, the perception that nurses shared responsibility with physicians was also found among physicians who did not believe that monitoring improved quality and among physicians who did not support continuation of the program.

## Discussion

Our study demonstrated that Israeli PCPs perceive nurses in a positive light and consider them key players in improving quality of care. While they perceive nurses as partners, they made minimal reference to the importance and contribution of nurses in the open-ended questions about improvement of quality indicators.

Various explanations could be proposed for this disparity. It is possible that in an era when there is a shortage of physicians, nurses have assumed a more significant role in health promotion and disease prevention, which the physicians have learnt to appreciate. The physicians’ perception might be a consequence of a long-standing cooperation with nurses, which has shaped a “new reality.” The disparity can also be attributed to the physicians’ understanding and recognition that health improvement is best managed through multidisciplinary work. This explains why, when asked about the nurses, the physicians praised them for their contribution. The implementation of the national quality monitoring program by the health plans has put pressure on physicians to achieve their targets (25), causing them to seek assistance from other parties, so it is only natural for the nurses to play an active role in helping to advance the quality monitoring.

However, in the wider context, when they were asked about changes in general, rather than specifically about nurses, the physicians hardly mentioned nurses, which may indicate that the nurses’ role has not yet been fully internalized or appreciated by the physicians.

Another explanation for the disparity between the physicians’ perception of the nurses as partners and the minimal mention of them in the open questions is that while the physicians did express their true appreciation for nurses as part of the quality team in the questions that had a direct bearing on nurses, when they were asked to discuss changes they had made or about areas where they could be helped with measurement, they focused on their own work and respected the traditional dividing line between the two professions. The physicians are likely to feel that they share responsibility with the nurses because the nurses are also part of the clinic staff heading the quality of care – nurses are assigned tasks that have to do with quality, such as making appointments for lists of patients, monitoring chronic patients, and promoting vaccinations for target populations. The physicians want the nurses to share responsibility because they have a heavy workload (25), and partnership with the nurses will justify the transfer of some of their work. However, when asked about changes they had made in their work or areas where the health plan could help them, they answered the question from the perspective of their own professional territory.

There are two aspects to the nurses’ involvement in quality indicators (1) assisting the physicians with the activity being monitored and (2) having responsibility for the indicators and making decisions regarding the patient’s treatment. In Table 2, we see that as many as 80% of the respondents agree or strongly agree that nurses “share full responsibility for performance” while only 30% agree or strongly agree that nurses responsibility is to “assist” the physician. It seems that physicians tend to believe that nurses have a broader role than simply to follow orders. Apparently, they are expected to get more involved and even exercise discretion. We are planning a study that will examine the nurses’ views on these issues.

### Work Practices in the Health Plans

In most aspects, the physicians held somewhat homogeneous views about the role of nurses. However, there were slight differences according to the type of employment, with salaried physicians more supportive of the idea that nurses should share the responsibility than self-employed physicians. One possible reason is that, in Israel, salaried physicians generally work in larger health plan clinics, with a staff that includes nurses, so that they are used to cooperating with them, find it natural and convenient, and have become used to it. In contrast, self-employed physicians often work in solo or small group practices, without a nurse in the team. Consequently, even if, in theory, they appreciate nursing work, in practice, it does not affect their work patterns and has not yet been fully internalized.

Some of the health plans have redesigned primary care management (26, 27), encouraging solo or group practices, even if they are independent, to introduce nurses and work with them as a team. This has brought physicians and nurses together in their work and led to greater interaction between them. The resultant direct dialog is likely to have affected the physicians’ perceptions about the nurses. Teamwork, respect, and a good relationship between physicians and nurses affect the quality of care of the patient (28, 29). It is therefore natural for the physicians’ positive attitude toward nurses to be expressed in their perception of the nurses’ contribution to improved care indicators. This might create an opportunity to re-define the position of nurses and strengthen their status.

The pattern of positive attitudes toward nurses, which was found in every group of physicians, may strengthen the physicians’ perception that nurses are indispensable in community care frameworks. These findings are compatible with attitudes known in the literature, which states that physician’s value nurses (especially NPs), and their interpersonal and training skills (23). Although physicians see nurses as their extender, and in primary care, they do not necessarily support in expanding nurses’ role (30), and nurses see their role as one of autonomous with physician backup when needed (23, 31).

## Conclusion

### The Role of the Nurses – Looking Ahead

Our study findings, which coincide with the current interest among policymakers in improving the quality of health care, create a unique opportunity to examine ways to include the nurses in monitoring and improving care. One of the major changes that could shape this shift would be to assign nurses’ direct responsibility for some of the clinical domains, particularly those that belong in their natural work environment (e.g., preventive medicine and some of the indicators for chronic patients).

We observed a willingness among physicians to share the responsibility for improving patient care with other staff members. Performance indicators reflect the quality of health care provided to the patient by various staff members. It is only natural, therefore, for physicians and nurses to be assessed and valued together as a single unit. The physicians’ responses indicate that we are ready for this step.

## Author Contributions

All authors made a substantial contribution to the conception and design of the work and to the analysis and interpretation of the data. RN-G, BR, and RW-M drafted the article, and all authors have reviewed it critically for intellectual content. All authors gave their final approval of the version published. All authors agreed to be accountable for all aspects of the work in ensuring that questions related to the accuracy or integrity of any part of the article are appropriately investigated and resolved.

## Conflict of Interest Statement

The authors declare that the research was conducted in the absence of any commercial or financial relationships that could be construed as a potential conflict of interest.

## References

[1] Blumenthal D Part 1: quality of care – what is it? N Engl J Med (1996) 335:891–4. 10.1056/NEJM199609193351213 8778612

[2] Chassin MR Loeb JM Schmaltz SP Wachter RM Accountability measures – using measurement to promote quality improvement. N Engl J Med (2010) 363:683–8. 10.1056/NEJMsb1002320 20573915

[3] Jakovljevic M Vukovic M Chen CC Antunovic M Dragojevic-Simic V Velickovic-Radovanovic R Do health reforms impact cost consciousness of health care professionals? Results from a nation-wide survey in the Balkans. Balkan Med J (2016) 33(1):8–17. 10.5152/balkanmedj.2015.15869 26966613 PMC4767315

[4] National Board of Health and Welfare and the Swedish Association of Local Authorities and Regions. Quality and Efficiency in Swedish Health Care – Regional Comparisons. (2013). Available from: http://www.socialstyrelsen.se/publikationer2013/2013-5-7

[5] Sutherland K Coyle N Quality in Healthcare in England, Wales, Scotland, Northern Ireland: An Intra-UK Chartbook. (2009). Available from: http://www.health.org.uk/sites/default/files/QualityInHealthcareInEnglandWalesScotlandNorthernIreland_IntraUKChartbook.pdf

[6] Jaffe DH Shmueli A Ben-Yehuda A Paltiel O Calderon R Cohen AD Community healthcare in Israel: quality indicators 2007-2009. Isr J Health Policy Res (2012) 1:3. 10.1186/2045-4015-1-3 22913466 PMC3415131

[7] Porath A Rabinowitz G Raskin Segal A Quality Indicators for Community Care in Israel, 2003-2005. (2006). Available from: http://healthindicators.org.il/wp-content/uploads/2014/08/Israel-quality-indicators-2003-2005-English1.pdf

[8] Aiken LH Clarke SP Sloane DM Sochalski JA Busse R Clarke H Nurses’ reports on hospital care in five countries. Health Aff (2001) 20(3):43–53. 10.1377/hlthaff.20.3.43 11585181

[9] Lacey SR Cox KS. Nursing: key to quality improvement. Pediatr Clin North Am (2009) 56(4):975–85. 10.1016/j.pcl.2009.05.004 19660639

[10] Mitchell P Waterworth S Embedding quality improvement change into nursing practice. Kai Tiaki Nurs Res (2012) 3:24–30.

[11] Van Loon A The changing professional role of community nurses. In: Kralick D Van Loon A, editors. Community Nursing in Australia. Carlton, VIC: Blackwell Publishing (2008). p. 315–30.

[12] Vukovic M Gvozdenovic BS Gajic T Stamatovic Gajic B Jakovljevic M McCormick BP. Validation of a patient satisfaction questionnaire in primary health care. Public Health (2012) 126(8):710–8. 10.1016/j.puhe.2012.03.008 22831911

[13] Cooper R. New directions for nurse practitioners and physician assistants in the era of physician shortages. Acad Med (2007) 82:825–79. 10.1097/ACM.0b013e31812f7939 17726384

[14] Vuković M Gvozdenović BS Ranković M McCormick BP Vuković DD Gvozdenović BD Can didactic continuing education improve clinical decision making and reduce cost of quality? Evidence from a case study. J Contin Educ Health Prof (2015) 35(2):109–18. 10.1002/chp.21272 26115110

[15] Curie V Relationship between quality of care, skill mix and nurse autonomy: literature review. J Adv Nurs (2005) 51:73–82. 10.1111/j.1365-2648.2005.03462.x 15941463

[16] Hines PA Yu KM. The changing reimbursement landscape: nurses’ role in quality and operational excellence. Nurs Econ (2009) 27:7–13. 19331307

[17] Swan BA. Making nursing-sensitive quality indicators real in ambulatory care. Nurs Econ (2008) 26(195–201):205. 18616060

[18] Augustin M Rustenbach SJ Debus S Grams L Münter KC Tigges W Quality of care in chronic leg ulcer in the community: introduction of quality indicators and a scoring system. Dermatology (2011) 2011(222):321–9. 10.1159/000328139 21757868

[19] Jansink R Braspenning J van der Weijden T Elwyn G Grol R. Primary care nurses struggle with lifestyle counseling in diabetes care: a qualitative analysis. BMC Fam Pract (2010) 11:41. 10.1186/1471-2296-11-41 20500841 PMC2889883

[20] Renders CM Valk GD Griffin SJ Wagner EH Eijk Van JT Assendelft WJ. Interventions to improve the management of diabetes in primary care, outpatient, and community settings: a systematic review. Diabetes Care (2001) 24:1821–33. 10.2337/diacare.24.10.1821 11574449

[21] Tschannen D Aebersold M Sauter C Funnell MM. Improving nurses’ perceptions of competency in diabetes self-management education through the use of simulation and problem-based learning. J Contin Educ Nurs (2013) 44:257–63. 10.3928/00220124-20130402-16 23565600

[22] Rosen B Nissanholtz-Gannot R From Quality Information to Quality Improvement – Interim Report: Summary and Analysis of Interviews with Health-Plan Managers. Jerusalem: Myers-Joint-Brookdale Institute (2010).

[23] Fletcher CE Baker SJ Copeland LA Reeves PJ Lowery JC. Nurse practitioners’ and physicians’ views of NPs as providers of primary care to veterans. J Nurs Scholarsh (2007) 39:358–62. 10.1111/j.1547-5069.2007.00193.x 18021137

[24] Mackay B. General practitioners’ perceptions of the nurse practitioner role: an exploratory study. N Z Med J (2003) 116:1170. 12658315

[25] Nissanholtz-Gannot R Rosen B Quality Monitoring Study Group. Monitoring quality in Israeli primary care: the primary care physicians’ perspective. Isr J Health Policy Res (2012) 1:1–13. 10.1186/2045-4015-1-26 22913311 PMC3472172

[26] Wilf-Miron R Kedem H Heiman A Goldman D Shem-Tov O Kokia E Redesign of community-based health services: the key to decreasing the quality gap. Harefuah (2008) 147:698–701. 18935758

[27] Wilf-Miron R Kokia E Gross R Redesigning Primary Care Services in Maccabi. Health Policy Monitor (2007). Available from: http://hpm.org/en/Surveys/Brookdale_Institute_-_Israel/10/Redesigning_primary_care_services_in_Maccabi.html

[28] Friese CR Manojlovich M. Nurse-physician relationships in ambulatory oncology settings. J Nurs Scholarsh (2012) 44:258–65. 10.1111/j.1547-5069.2012.01458.x 22812518 PMC3432717

[29] Ponte PR Gross AH Milliman-Richard YJ Lacey K. Interdisciplinary teamwork and collaboration: an essential element of a positive practice environment. Annu Rev Nurs Res (2010) 2010(28):159–89. 10.1891/0739-6686.28.159 21639027

[30] Donelan K DesRoches CM Dittus RS Buerhaus P. Perspectives of physicians and nurse practitioners on primary care practice. N Engl J Med (2013) 368:1898–906. 10.1056/NEJMsa1212938 23675658

[31] Kowalczyk L CVS Seeks to Open Clinics in Its Stores – Would be First in State: Health Officials Cautious. Boston Globe (2007). Available from: http://archive.boston.com/business/articles/2007/05/02/cvs_seeks_to_open_clinics_in_its_stores/

